# Supplementary material for: SyNDI: synchronous network data integration framework
Source: BMC Bioinformatics. 2018 Nov 6;19:403. doi: 10.1186/s12859-018-2426-5 (PMC6219086; doi:10.1186/s12859-018-2426-5)
Supplement: Supplementary file 5 — Files of the example presented in Additional file 4. This additional file is a zip package that contains all files used in Additional file 4. Detailed descriptions of each of these files are presented in a README file included in this zip package. (GZ 30411 kb) [file 12859_2018_2426_MOESM5_ESM.gz › SyNDITools/Meme2Fimo/meme_4.9.0_4.tar.gz!/meme_4.9.0/doc/meme2meme.html]

meme2meme


# meme2meme

**Usage:** `meme2meme [options] [<meme file>]+`

**Description:**

Convert and merge multiple MEME formatted files into a single minimal
meme format file.

**Input:**

Multiple meme files. The new meme html format is supported but the old html format is not.

**Options:**

-consensus
:   numeric names are swapped for an IUPAC consensus; default: use existing names

-numbers
:   use numbers instead of strings for motif names; default: use existing ID

-bg <background file>
:   file with background frequencies of letters; default: use first file background

-logodds
:   print log-odds matrix as well as frequency matrix; default: frequency matrix only

-url <website>
:   website for the motif if it doesn't have one already; The motif name is substituted for MOTIF\_NAME; default: use existing url

-forceurl
:   Existing urls are ignored

**Output:**

Writes MEME format to standard output.
